# Liver metastasis originating from colorectal cancer with macroscopic portal vein tumor thrombosis: a case report and review of the literature

**DOI:** 10.1186/1752-1947-4-382

**Published:** 2010-11-26

**Authors:** Yoshito Tomimaru, Yo Sasaki, Terumasa Yamada, Kunihito Gotoh, Shingo Noura, Hidetoshi Eguchi, Isao Miyashiro, Masayuki Ohue, Hiroaki Ohigashi, Masahiko Yano, Osamu Ishikawa, Shingi Imaoka

**Affiliations:** 1 Department of Surgery, Osaka Medical Center for Cancer and Cardiovascular Diseases, Osaka, Japan; 2 Department of Surgery, Graduate School of Medicine, Osaka University, Osaka, Japan; 3 Department of Surgery, Yao Municipal Hospital, Osaka, Japan

## Abstract

**Introduction:**

Macroscopic tumor thrombi occupying the main portal branch are rarely seen in patients with liver metastasis.

**Case presentation:**

A 55-year-old Japanese man who had previously undergone surgery for adenocarcinoma of the ascending colon presented with a metastatic liver tumor accompanied by a macroscopic tumor thrombus in the right portal branch. Right lobectomy and removal of the tumor thrombus were performed, and the liver metastasis and tumor thrombus were successfully resected. Histopathological examination of the liver tumor revealed adenocarcinoma, consistent with that of the previous colon cancer, confirming that the liver tumor was a metastasis from the colon cancer. Our patient remains well without recurrence at 51 months after the liver surgery.

**Conclusion:**

The prognosis of patients with liver metastasis accompanied by a portal vein tumor thrombus remains unknown, but, considering several previous reported cases together with our case report, a better prognosis may be expected if the tumor is successfully removed by anatomical liver resection.

## Introduction

Portal vein tumor thrombosis (PVTT) is associated with hepatocellular carcinoma (HCC), with a reported incidence of PVTT of 30% to 70% [1-3]. A recent pathological study of metastatic liver cancer originating from colorectal cancer found microscopic tumor invasion in the intra-hepatic portal vein to be a relatively common finding in addition to HCC [4,5]. However, macroscopic tumor thrombi occupying the main portal branch are rare in patients with liver metastasis [6,7], including that from colorectal cancer (Table 1) [8-14].

**Table 1:** Previously reported cases with macroscopic portal vein thrombus (PVTT) from successfully resected colorectal cancers

**Case no**.	Reference	Age and gender	Synchronous or metachronous	Location of primary tumor	Histology	Stage	Interval from colorectal resection to diagnosis of PVTT, months	Size of liver metastasis, mm	Location of liver metastasis	Location of PVTT	Survival after removal of PVTT, months	Prognosis
1	Tanaka *et al. *[8]	59/M	Synchronous	Sigmoid	Mod	T3N1	-	Unknown	S8, left	Left PV	11	Alive, recurrence
2	Tanaka *et al. *[8]	54/M	Metachronous	Rectum	Mod	T4N2	12	70	S2/3	Left PV	21	Alive, no recurrence
3	Tanaka *et al. *[8]	60/M	Metachronous	Transverse	Poor	T3N2	4	25	S7	Right PV	31	Alive, no recurrence
4	Tanaka *et al. *[8]	63/F	Metachronous	Sigmoid	Well	T3N0	47	55	S6	Posterior PV	55	Alive, no recurrence
5	Tanaka *et al. *[8]	62/F	Metachronous	Descending	Mod	T3N1	11	-	-	Right PV	102	Alive, no recurrence
6	Lee *et al. *[10]	28/M	Synchronous	Sigmoid	Muc	Unknown (N+)	-	40	S2/3	Left PV branch	1.5	Alive, recurrence
7	Sugiura *et al. *[11]	39/F	Metachronous	Transverse, rectum	Well	Unknown	141	Unknown (huge)	S4/5/6/7/8	Left PV	24	Alive, no recurrence
8	Urahashi *et al. *[12]	57/M	Metachronous	Transverse	Mod	T3N1	< 24	40	S6/7	Main PV	11	Died, recurrence
9	Urahashi *et al. *[12]	51/M	Metachronous	Transverse	Mod	T3N2	< 24	145	S7/8	Anterior PV	9	Died, recurrence
10	Urahashi *et al. *[12]	54/M	Metachronous	Rectum	Well	T3N2	< 24	35	S3	Left PV	36	Died, recurrence
11	Urahashi *et al. *[12]	70/F	Metachronous	Ascending	Mod	T3N1	< 24	-	-	Main PV	6	Died, recurrence
12	Urahashi *et al. *[12]	45/F	Metachronous	Descending	Mod	T3N2	< 24	60	S5, S6, S8	Main PV	10	Died, recurrence
13	Oikawa *et al. *[13]	55/F	Synchronous	Rectosigmoid	Muc	T3N1	-	100	S6/7, left	Posterior PV	9	Died, recurrence
14	Matsumoto *et al. *[14]	58/M	Metachronous	Rectosigmoid	Mod	T3N0	6	-	-	Left PV	66	Alive, no recurrence
15	Present case	55/F	Metachronous	Ascending	Mod	T4N1	13	28	S8	Right PV	51	Alive, no recurrence

Mod = moderately differentiated adenocarcinoma; poor = poorly differentiated adenocarcinoma; PV = portal vein; PVTT = portal vein tumor thrombosis; well = well differentiated adenocarcinoma; muc = mucinous adenocarcinoma.

We report on a case of liver metastasis from colon cancer with macroscopic tumor thrombi in the right portal branch. Herein, we describe the case and review the literature for liver metastases from colorectal cancer accompanied by macroscopic portal vein tumor thrombi.

## Case presentation

A 55-year-old Japanese man underwent a right hemicolectomy in our hospital for a tumor in the ascending colon.. He did not have any inherited or acquired thrombophilic predispositions. The tumor was histopathologically diagnosed as moderately differentiated adenocarcinoma, and staged as IIIB (T4N1M0), according to the TNM (tumor, nodes, metastasis) classification [15]. Tumor markers including carcinoembryonic antigen (CEA) and carbohydrate antigen 19-9 (CA19-9) were all within normal limits before the operation. During follow-up in our outpatient clinic, our patient received adjuvant systemic chemotherapy for six months.

**Figure 1:**
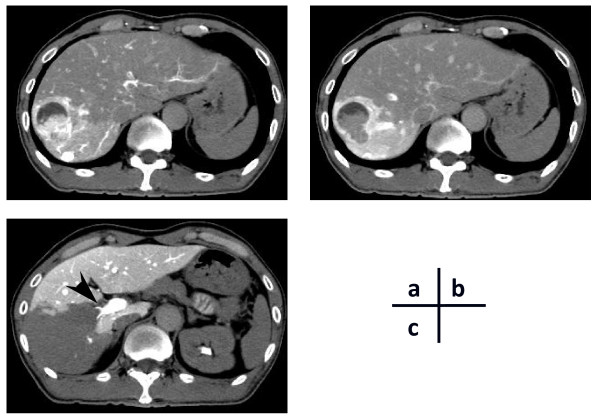
**Computed tomography arteriography (CTA) of the liver tumor in the early phase (A) and the delayed phase (B)**. **C)** Computed tomography during arterial portography (CTAP) showing a portal vein tumor thrombus (arrow) and a perfusion defect in the entire right lobe.

**Figure 2:**
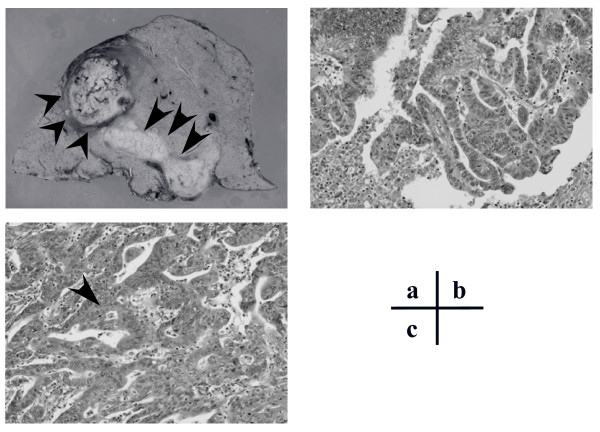
**A) Macroscopic view of the resected liver including the metastatic liver tumor (arrowheads) and the tumor thrombus in the right portal vein (arrows)**. Histopathological findings of the metastatic liver tumor **(B)** and primary colon cancer **(C)** showing moderately differentiated adenocarcinoma.

Despite the adjuvant treatment, abdominal computed tomography (CT) 13 months after surgery showed a liver tumor in segment 8 based on Couinaud's classification [16]. Our patient was subsequently readmitted to our hospital for full diagnosis and treatment of the liver tumor. Hepatitis B surface antigen, hepatitis B core antibody, and hepatitis C antibody test results were negative. Tumor markers including CEA, CA19-9, α-fetoprotein, and protein induced by vitamin K absence or antagonist II, were all within normal limits. CT arteriography (CTA) showed a tumor of approximately 25 mm in diameter consisting of two components: an apparently solid part and a cystic component. The solid component of the tumor was enhanced in the early phase of the CTA and was washed out in the delayed phase, a pattern compatible with HCC (Figure 1A). However, based on the cystic component, the tumor was also suspected to be a cystadenocarcinoma. The right portal vein was not visible on portography, but CT during arterial portography (CTAP) revealed defective portal perfusion in the whole right lobe of the liver (Figure 1C). This finding was suggestive of PVTT. Endoscopic retrograde cholangiography was performed to differentiate cystadenocarcinoma connected to a biliary duct. However, no specific findings of biliary carcinoma were noted and the collected bile sample was cytologically negative. For preoperative differential diagnosis of the tumor, echo-guided biopsy was performed. The biopsy revealed that the liver tumor was a liver metastasis from the colon cancer. With a preoperative diagnosis of liver metastasis from colon cancer, laparotomy was performed. Neither peritoneal dissemination nor hilar lymph node metastasis was detected. The liver tumor, measuring 28 × 25 mm in size, was located in segment 8, while PVTT was located in the right portal vein in direct communication with the liver tumor. Our patient underwent a right lobectomy (Figure 2A). The resected tumor, which had a fibrotic capsule, macroscopically resembled HCC. The cystic component observed on preoperative examination was not detected in the resected specimen. Histopathology of the resected liver tumor and PVTT revealed a moderately differentiated adenocarcinoma (Figure 2B). The histopathological findings from the resected tumor were similar to the previously resected ascending colon cancer. Based on the similarity, the final diagnosis for the liver tumor was a liver metastasis from the ascending colon cancer accompanied by macroscopic PVTT in the right portal branch. Histopathological infiltration into the endothelial layer of the portal vein was not seen. All resected margins were free from cancer. Postoperatively, our patient agreed to receive adjuvant chemotherapy. Our patient remains healthy, with no evidence of recurrence 51 months after the hepatectomy.

## Discussion

Microscopic tumor invasion into the intra-hepatic portal vein is detected in about 20% of cases with liver metastasis from colorectal cancer [4]. However, our review of previously reported cases revealed few instances of PVTT in the main portal branch [8-14]. In fact, the reported incidence of macroscopic PVTT similar to that observed in our case report is 2.8% (4 of 142) [9]. From January 1990 to December 2008, 231 patients underwent resection of liver metastases from primary colorectal cancer in our hospital. Of these patients, only our patient's case showed macroscopic PVTT (0.4%).

Macroscopic examination of the resected tumor in our patient did not show the preoperatively detected cystic component of the tumor. It is possible that necrotic fluid, having filled the cystic component, was absorbed and thus replaced by the tumor before removal. The resected liver tumor and PVTT macroscopically resembled HCC, which commonly develops tumor thrombi and expansive growth in the portal vein and in the hepatic vein [17]. The capsule formation of HCC is possibly the result of mechanical compression or high inner pressure from the expansive tumor growth, thus it is also feasible that tumor thrombi might extend into the portal vein via a pressure gradient mechanism [18]. In contrast, liver metastases from colorectal cancer are generally less commonly surrounded by a capsule compared to HCC, with one study detecting encapsulated liver metastases from colorectal cancer in only 20% of cases [19]. The resected tumor in our patient, which was encapsulated, also resembled HCC in this point of the capsule formation. This resemblance to HCC may suggest that the PVTT in this case might have also expanded into the portal vein through a pressure gradient mechanism, as in HCC.

Table 1 summarizes 15 reported cases of liver metastasis from colorectal cancer with macroscopic PVTT, including our patient. No specific clinical features typified patients with colorectal liver metastasis and PVTT with respect to age, gender, or the primary tumor site. With regard to the stage of the primary colorectal cancer, all the primary colorectal lesions recorded were divided into T3 or T4 according to the TNM classification [15], and lymph node metastasis was found in most of the cases (12 of 14, 86%). In 12 of the 15 cases (80%), liver metastasis was accompanied by PVTT, and the liver tumor was relatively large (60 ± 37 mm; range, 25 to 145 mm). PVTT was found metachronously in 12 patients, and synchronously with the primary tumor in the remaining three patients. Although Matsumoto *et al. *[14] suggested that survival after the operation of PVTT from colorectal cancer might depend on whether the PVTT had developed synchronously or metachronously, this suggestion seems not to be applied to the review in the present study. With regards to the liver tumor, anatomical liver resection was performed in all 15 patients. The one-year, three-year and five-year overall survival rates in the 15 cases after operation for PVTT were 64.3%, 51.4%, and 51.4%, respectively. Since this analysis was performed only in a limited number of patients, specifically successful cases, the analysis did not allow a precise general prognosis to be determined for metastatic liver tumor with PVTT. However, even if the aforementioned success bias was taken into consideration, this outcome seems to be relatively good. In general, anatomical liver resection is not usually employed for colorectal liver metastasis in contrast to HCC [20-22]. However, considering that colorectal liver metastasis with PVTT is likely to spread along the portal tributaries as in HCC, it may be speculated that anatomical liver resection, which is suitable for such liver metastasis, contributes to the favorable prognosis for colorectal liver metastasis with PVTT, as suggested by some investigators [9,10,14]. Today, some treatment options for colorectal liver metastasis have been established including surgery, ablation therapy, hepatic arterial infusion chemotherapy, and systemic chemotherapy, but there is no consensus for the treatment for colorectal liver metastasis accompanying PVTT. This successful case is not enough to conclude that surgery is the best treatment option for such liver metastasis, but we suggest at least that macroscopic PVTT is not a contraindication to liver surgery.

## Conclusion

Our patient had a successfully resected liver metastasis from colorectal cancer with macroscopic PVTT. The prognosis of patients with such PVTT remains unclear, but from previous reports it would appear a better prognosis can be expected if the tumor is successfully resected by anatomical liver resection.

## Competing interests

The authors declare that they have no competing interests.

## Authors' contributions

YT researched the case, reviewed the literature, and was a major contributor to preparation of the manuscript. YS was responsible for the research and review. TY, KG, SN, HE, IM, and MO supported the preparation of the manuscript. HO, MY, OI, and SI prepared the final version of the manuscript. All the authors read and approved the final manuscript.

## Consent

Written informed consent was obtained from the patient for publication of this case report and any accompanying images. A copy of the written consent is available for review by the Editor-in-Chief of this journal.
